# Psychological distress and health behaviours in people living with and beyond cancer: a cross-sectional study

**DOI:** 10.1038/s41598-024-66269-6

**Published:** 2024-07-04

**Authors:** Natalie Ella Miller, Phillippa Lally, Rana Conway, Andrew Steptoe, Philipp Frank, Rebecca J. Beeken, Abi Fisher

**Affiliations:** 1 Department of Behavioural Science and Health, Institute of Epidemiology and Health Care, University College London, 1-19 Torrington Place, Gower Street, London, UK; 2 Department of Psychological Sciences, University of Surrey, Guildford, Surrey GU2 7XH UK; 3 UCL Brain Sciences, University College London, 149 Tottenham Court Rd, London, W1T 7BN UK; 4 Leeds Institute of Health Sciences, University of Leeds, Leeds, LS2 9JT UK

**Keywords:** Psychological distress, Health behaviours, Cancer survivorship, WCRF recommendations, Cancer, Psychology, Cancer

## Abstract

This study aimed to examine whether psychological distress was cross-sectionally associated with meeting World Cancer Research Fund (WCRF) recommendations in people living with and beyond cancer. Participants were adults living with and beyond breast, prostate and colorectal cancer, participating in the baseline wave of the Advancing Survivorship after Cancer Outcomes Trial (ASCOT). Anxiety/depression was assessed using the EQ-5D-5L and dichotomised into any/no problems. WCRF recommendations were assessed via pedometers, 24-h dietary recalls, self-reported alcohol intake (AUDIT-C), and self-reported smoking status. Participants were categorised as meeting WCRF recommendations using the following cut-offs: average daily steps (≥ 10,000/day), average weekly aerobic steps (≥ 15,000/day), fruit and vegetables (≥ 400 g/day), fibre (≥ 30 g/day), red meat (< 500 g/week), processed meat (0 g/day), high calorie food (fat ≤ 33% of total daily energy intake and free sugar ≤ 5% of total daily energy intake), alcohol (≤ 14 units/week) and smoking (non-smoking). A composite health behaviour risk index (CHBRI) was calculated by summing the number of WCRF recommendations met (range: 0–9). Among 1348 participants (mean age = 64 years (SD = 11.4)), 41.5% reported anxiety/depression problems. The mean CHBRI score was 4.4 (SD = 1.4). Anxiety/depression problems were associated with lower odds of meeting WCRF recommendations for average daily steps (odds ratio (OR) = 0.73; 95% CI 0.55, 0.97), but not for any other health behaviour. Psychological distress is associated with lower adherence to WCRF recommendations for physical activity in people living with and beyond cancer. Physical activity may be a mechanism linking psychological distress and poorer outcomes among people living with and beyond cancer, and this should be explored in longitudinal studies.

## Introduction

The number of people living with and beyond cancer (LWBC) (i.e. people diagnosed with cancer at any point in their lifetime, who are either currently undergoing treatment or have completed treatment^1^) in the United Kingdom (UK) is continually rising due to increases in cancer incidence and, concomitantly, higher survival rates^2^. The growing number of people LWBC highlights the importance of understanding psychosocial factors that influence survival, and, ultimately, to develop supportive interventions aimed at improving outcomes.

The term psychological distress refers to symptoms of depression or anxiety^3^. A cancer diagnosis can be a highly distressing life event^3^. Cancer is associated with fear relating to pain, recurrence and death^4^, and can contribute to stigma^5^, relationship issues^6^, employment and financial difficulties^7^ and body image concerns^8^. The prevalence of psychological distress is high among people LWBC. A cross-sectional study of 10,153 people with cancer found that the prevalence of anxiety and depression was 19% and 12.9% for clinical levels, and 22.6% and 16.5% for subclinical levels, respectively^9^. Research also shows that the prevalence of depression is more than five times higher in people LWBC compared to the general population^10^. Higher levels of psychological distress among people LWBC are associated with poorer outcomes, such as poorer quality of life^11^, lower adherence to treatments^12^, and ultimately poorer survival^13^.

Healthy behaviours such as eating a healthy diet, engaging in regular physical activity and low alcohol consumption are associated with improved outcomes among people LWBC^14^. For instance, a meta-analysis of 117 cohort studies including 209,597 people LWBC found that a prudent diet (i.e. a diet low intake of red and processed meats, sugary foods and refined grains) was associated with a 24% lower risk of recurrence and overall mortality^15^. Another meta-analysis of eight randomised controlled trials found that exercise was associated with a 48% lower risk of cancer recurrence in people affected by cancer^16^. There is also evidence from cohort studies that higher post-diagnosis alcohol intake is associated with greater risk of cancer recurrence and mortality^15,17,18^. Most of this evidence comes from people affected by breast, prostate and colorectal cancer^19^—Three of the most commonly diagnosed cancers^20^. Consequently, the World Cancer Research Fund (WCRF) and the American Institute of Cancer Research (AICR) advise that people LWBC follow their cancer prevention recommendations, which are to participate in at least 150 min of moderate-to-vigorous physical activity per week; limit sedentary behaviour; consume plenty of wholegrains, fruits, vegetables and legumes; avoid sugary drinks and processed foods high in fat, starches or sugars; limit consumption of red meat; avoid processed meat; avoid alcohol; and maintain a healthy weight^21^. The WCRF and AICR also recognise the importance of avoiding smoking to reduce cancer risk.

Numerous theories of health behaviour such as the Social Ecological Model recognise that psychological factors can influence health behaviours^22^. Several observational studies have found that depression and anxiety are inversely associated with physical activity among people LWBC^23–25^. One cross-sectional study found that depression is inversely associated with meeting the National Physical Activity Guidelines of Australia (NPAGA) (150 min of moderate-to-vigorous physical activity per week) in 638 men with prostate cancer^26^. However, most of these prior studies relied on small sample sizes (*N* < 650) and used self-report measures of physical activity. Self-reported physical activity is prone to recall bias and can overestimate levels of activity compared to device-based measures^27^. A cross-sectional study assessing physical activity using accelerometery found that meeting physical activity guidelines was associated with fewer anxiety symptoms^28^. However, this study was conducted among a small sample of 180 people with colon cancer. Fewer studies have examined the association between psychological distress and diet quality, and findings are mixed^29–31^. Furthermore, most of these studies assessed dietary intake using questionnaires, which are not as accurate as more comprehensive measures of dietary intake such as 24 h recalls. Some studies have shown that greater psychological distress is associated with greater alcohol consumption and smoking in people LWBC^32–34^. To date, no studies have examined whether psychological distress is associated with meeting recommended guidelines for diet/alcohol intake among people LWBC. Furthermore, few studies have assessed whether psychological distress is associated with the total number of WCRF recommendations met among people LWBC, and these have small sample sizes^35,36^.

Therefore, this study aimed to examine whether anxiety/depression is associated with (1) the total number of WCRF recommendations met and (2) meeting WCRF guidelines for individual health behaviours among a large sample people affected by breast, prostate and colorectal cancer. Physical activity was assessed objectively using pedometers, and diet was assessed via 24 h recalls.

## Methods

### Data

This cross-sectional study used data collected as part of the baseline assessment for the advancing survivorship after cancer outcomes trial (ASCOT)^37^. ASCOT is a randomised controlled trial of a health behaviour intervention for people LWBC.

### Participants

Participants were recruited from ten NHS trusts across London and Essex. These hospital sites were asked to send out a ‘Health and Lifestyle after Cancer’ survey to all patients diagnosed with breast, prostate and colorectal cancer between 2012 and 2015. However, hospitals did not always correctly identify diagnosis dates, so ethical approval was obtained to include individuals diagnosed outside of these dates. Patients completed the questionnaire on paper or online and then returned it to the research team. Of 13,546 surveys sent, 5835 were returned (response rate = 42.8%). At the end of the questionnaire, patients had the option to leave their contact details to learn more about a trial of a lifestyle intervention Individuals who expressed interest were assessed for eligibility. A total of 3354 individuals indicated interest (57.5% of surveys returned), of which 1348 were eligible to participate (40.1% of those who expressed interest). Individuals were eligible to participate if they were aged 18 years and over, were diagnosed with non-metastatic breast, prostate or colorectal cancer (Stage I-III—Primarily assessed by patient report during screening), and were not currently receiving active anti-cancer treatment (with the exception of oral anti-cancer treatments taken at home). Eligible participants were then provided with the full trial information and asked to provide informed consent to participate. National cancer registry data were collected for the majority of participants and when this was received it was discovered that 14 participants had Stage IV cancer at diagnosis and 28 had Stage 0 cancer, but these participants were still included in analyses. Ethical approval for the ASCOT was obtained through the National Research Ethics Service Committee South Central—Oxford B (reference number 14/SC/1369), and all methods were performed in accordance with the relevant guidelines and regulations. All participants provided informed consent to participate.

### Measures

#### Physical activity

Physical activity was assessed using an Omron pedometer (Omron, Kyoto, Japan) with the count reader covered^38^. Omron pedometers have established validity and reliability at different walking and running speeds^38,39^. The method for how the pedometer data were processed is described in detail elsewhere^40^. Participants were asked to wear the pedometer all day for six days, on their waist or in their pocket, except when showering, bathing, swimming, doing water sports, or doing contact sports. Participants were also asked to complete a log-book indicating the dates they wore the pedometer, the time they put the pedometer on and took it off each day, and any physical activity they performed when they took the pedometer off. The pedometer data was cleaned using the log-books so that physical activity reported to have been performed when the pedometers were off was included. If data were not available for two days or more, then pedometer data were discarded to ensure there were enough days to provide a meaningful estimate of average daily steps^41^.

Data from pedometers were uploaded using the Omron software Bi-link gateway (Omron). This provided the number of average daily steps, and the number of average weekly steps classified as aerobic (steps walked at a pace of 60 steps/min or higher for bouts of 10 min or more)^42^. For average daily steps, a cut-off of 10,000 was used to denote meeting physical activity guidelines^43^. For weekly aerobic steps, a cut-off of 15,000 was used to indicate meeting physical activity guidelines. This cut-off was chosen based on the assumption that when participants walk at an aerobic pace, they on average walk at a pace of 100 steps per minute^44^.

#### Diet

Diet was assessed using 24 h dietary recalls. The process for collecting and processing the recalls is described in detail elsewhere^45^. In brief, participants used Myfood24^®^ online software to search a database for food and drink items they have consumed the previous day, select the most appropriate option, and determine portion size by selecting one of a range of pictures or by inputting data from household measures or weights. Participants were asked to complete recalls on one weekday and one weekend day.

Participants were sent letters with dates they were due to complete their weekday and weekend day recalls. On the day of their first scheduled recall, participants were sent emails with instructions on how to self-complete their recall and a link to Myfood24^®^. Participants who did not use email were contacted by telephone by a researcher who collected dietary information and inputted this into Myfood24^®^ on their behalf. These individuals were sent a booklet before the call containing food portion images taken (with permission) from A Photographic Atlas of Food Portion Sizes to help with portion size estimation^46^. If participants had any questions or queries when completing their recalls, or if researchers noticed any unusual data entries, participants were contacted to resolve issues.

When the recalls were complete, data from Myfood24^®^ were exported as an Excel file, and cleaned by experienced researchers who were registered dietitians or individuals with a post-graduate qualification in nutrition. Any unusually small or large data entries were inspected and only changed if two researchers agreed this was an error. After cleaning the dietary data, weighted average daily intake was calculated, with the weekday recall given a weighting of five and the weekend recall a weighting of two. Percentage energy from fat was calculated as 9 kcal/g, and percentage energy from sugar was calculated as 3.75 kcal/g.

To assess adherence to the five WCRF recommendations for diet, the following cut-offs were used to denote adherence: (1) fruit and vegetables – at least five portions (400 g) per day^21^, (2) fibre – at least 30 g per day^21^, (3) red meat – less than 500 g per week^21^, (4) processed meat – 0 g per day^21^, and (5) high calorie food – total calories from fat ≤ 33% of total energy intake^47^ and free sugar percentage of daily calories ≤ 5% of total energy intake^48^.

#### Alcohol

Alcohol consumption was assessed using two questions, adapted from the AUDIT alcohol consumption questions^49^. The first item was “How often do you have a drink containing alcohol?” with response options “never”/”monthly or less”/”2–4 times per month”/”2–3 times per week”/”4–5 times per week”/”every day”. The second item was “how many units of alcohol do you drink on a typical day when you are drinking?” with response options “never”/“1–2”/“3–4”/“5–6”/“7–9”/“10 + ”. These two responses were converted to numerical scores and multiplied to estimate the total number of units consumed on average per week. The total score ranged from 0 to 70 units per week. Given that national UK guidelines for alcohol consumption recommend not drinking more than 14 units of alcohol per week^50^, this was used as the cut-off to denote meeting versus not meeting recommendations.

#### Smoking

Smoking status was assessed using a single item from the Health Survey for England which indicated whether participants were a current smoker or non-smoker^51^. Smokers were classified as not meeting national guidelines for smoking whereas non-smokers were classified as meeting guidelines.

#### CHBRI index

The composite health behaviour risk index (CHBRI) was calculated based on nine health behaviours recommended by the WCRF for people LWBC (average daily steps, weekly aerobic steps, fruit and vegetables, fibre, red meat, processed meat, high calorie food, alcohol and tobacco). Table 1 shows the cut-offs used to determine whether participants were/were not meeting the guidelines. Participants were given a score of 1 if they were meeting guidelines, and a score of 0 if they were not. To calculate the CHBRI, these scores for each of the nine behaviours were summed. The CHBRI ranged from 0 (not meeting any recommendations) to 9 (meeting all recommendations).

**Table 1:** Health behaviour cut-off points determining whether ASCOT participants are meeting WCRF recommendations.

Behaviour	Meeting (score = 1)	Not meeting (score = 0)
Daily physical activity	≥ 10,000 average steps/day	< 10,000 average steps/day
Fitness	≥ 15,000 weekly aerobic steps	< 15,000 weekly aerobic steps
Fruit and vegetables	≥ 400 g/day (one portion = 80 g)	< 400 g/day
Fibre	≥ 30 g per day	< 30 g/day
Red meat	< 500 g/week	≥ 500 g/week
Processed meat	0 g/day	> 0 g/day
High calorie food	Fat: ≤ 33% of total energy intake Sugar: free sugar percentage of daily calories ≤ 5% of energy intake	Fat: > 33% of total energy intake Sugar: free sugar percentage of daily calories > 5% of energy intake
Alcohol	≤ 14 units/week	> 14 units/week
Tobacco	Non-smoker	Smoker

### Psychological distress

Psychological distress was assessed using the anxiety/depression dimension of the five-level EuroQol-5D questionnaire (EQ-5D-5L)^52^. The EQ-5D-5L has been validated for use in people LWBC^53^. The anxiety/depression dimension consists of one item asking participants to report if they were “not”/”slightly”/”moderately”/”severely”/”extremely” anxious or depressed on that day and is scored from 1 (no problems) to 5 (severe problems). In this study, due to issues with skewness, anxiety/depression scores were dichotomised into no problems (score = 1) versus any problems (score = 2–5). This method of dichotomising EQ-5D-5L index scores has been used previously in large samples of people LWBC^54,55^.

### Covariates

Participants reported their age in years, sex (male/female), ethnicity (dichotomised into white/non-white due to small numbers in some ethnic groups) and marital status (dichotomised into married/not married) and highest level of education (none/GCSE or vocational/A level/degree or above). Participants were also asked to report if they had any of the following comorbidities: osteoporosis, diabetes, asthma, stroke, Parkinson’s disease, Alzheimer’s disease or dementia, lung disease, arthritis, angina, heart attack, heart murmur, irregular head rhythm, any other heart problem or hypertension. The total number of comorbidities participants reported was summed. Height and weight were self-reported, and body mass index was calculated using the formula weight(kg)/(height(m))^2^.

Cancer type, stage at diagnosis, and date of diagnosis were all self-reported and if consent was given, these data were also provided by the National Cancer Registration and Analysis Service (NCRAS). NCRAS data were used if available, but if not available, then self-report data were used. For some people, NCRAS data suggested that they had been diagnosed with another cancer since their breast/prostate/colorectal cancer diagnosis. Hence, in this study, cancer type was categorised into most recent diagnosis of breast, prostate, colorectal, or breast/prostate/colorectal plus one other. The number of days between this most recent cancer diagnosis and baseline assessments was calculated. Participants also self-reported on the treatment received for their most recent cancer, which was categorised into surgery only, surgery plus any other treatment, other treatments, and no treatment/active surveillance.

### Analysis

#### Missing data

Multiple imputation (MI) by chained equations was used to impute missing data on predictors, outcomes and covariates given recommendations to impute all three^56^. Twenty imputed datasets were generated and pooled using Rubin’s rules^57^.

#### Descriptive statistics

Descriptive statistics for the observed and imputed datasets were calculated. Means and standard deviations (SDs) were calculated for continuous variables, and frequencies and percentages were computed for categorical variables.

#### Main analyses

Multiple linear regression was conducted to assess the association between anxiety/depression and the CHBRI index. Logistic regression was conducted to assess associations between anxiety/depression and meeting WCRF recommendations for each health behaviour. All assumptions were tested for and met. There was no evidence of multicollinearity (variance inflation factors were less than 10 and tolerance values greater than 0.2). Analyses were adjusted for all covariates. The results for binary outcomes (meeting WCRF recommendations/not) are reported as adjusted odds ratios (ORs) and 95% confidence intervals (CIs). The results for continuous outcomes (CHBRI) are reported as beta (B) coefficients and 95% confidence intervals. Two models were run for each analysis. Model 1 included age and sex, and Model 2 included age, sex, ethnicity, marital status, highest level of education, total number of comorbidities, cancer type, cancer stage, treatment, and time between cancer diagnosis and baseline assessments. Stata version 18.0 was used for all analyses.

#### Sensitivity analyses

Two sensitivity analyses were conducted. First, the analyses were repeated on a sample of participants with no missing data on the exposure, outcome and covariates. Second, the analyses were repeated with body mass index added to Model 2, as it was uncertain if body mass index was on the causal pathway (e.g. depression—> weight gain—> lower fitness behaviours) or acted as a confounder.

## Results

### Descriptive statistics

Sample characteristics are reported in Table 2. A comparison of the baseline characteristics of the sample in the observed and imputed data is shown in Supplementary Table 1. Of the 1348 participants included in this study, 520 (38.6%) were male and 828 (61.4%) were female. The mean age of participants was 64 years (SD = 11.4). A total of 552 individuals (42%) reported anxiety/depression problems. The proportion of individuals meeting WCRF guidelines was 10.8% for average daily steps, 28.5% for average weekly aerobic steps, 45.9% for daily fruit and vegetable intake, 9.9% for daily fibre intake, 87.4% for weekly red meat intake, 49.9% for daily processed meat intake, 4.1% for high calorie food, 86.6% for units of alcohol per week, and 96.3% for smoking. The mean CHBRI score of the sample was 4.4 (SD = 1.4).

**Table 2:** Participant characteristics at baseline.

	Mean (SD)/*n* (%)
Age, years, *N* = 1345	64.2 (11.4)
Sex, *N* = 1348	
Male	520 (38.6)
Female	828 (61.4)
Ethnicity, *N* = 1342	
White	1242 (92.5)
Non-white	100 (7.5)
Marital status, *N* = 1347	
Married	957 (71.1)
Not married	390 (29.0)
Highest education, *N* = 1254	
None	226 (18.0)
GCSE/vocational	412 (32.9)
A level	174 (13.9)
Degree or above	442 (35.2)
Total number of comorbidities, *N* = 1348	0.8 (1.0)
0	643 (47.7)
1	436 (32.3)
2	176 (13.1)
3	70 (5.2)
4 +	23 (1.6)
Cancer type, *N* = 1348	
Breast	711 (52.7)
Prostate	352 (26.1)
Colorectal	238 (17.7)
Breast/prostate/colorectal + one other	47 (3.5)
Cancer stage, *N* = 1136	
0	28 (2.5)
1	439 (38.6)
2	426 (37.5)
3	229 (20.2)
4	14 (1.23)
Treatment, *N* = 1321	
Surgery only	264 (20.0)
Surgery and at least one other	780 (59.1)
Any combination of other treatment	208 (15.8)
No treatment or active surveillance	69 (5.2)
Time between cancer diagnosis and baseline assessments, days, *N* = 1348	1070.9 (382.1)
BMI, *N* = 1273	27.0 (4.7)
EQ-5D-5L anxiety/depression severity, *N* = 1329	
No problems	777 (58.5)
Problems	552 (41.5)
Average daily steps, *N* = 1236	5905 (3287)
Meeting guidelines	133 (10.8)
Not meeting guidelines	1103 (89.2)
Average weekly aerobic steps, *N* = 1236	11,539 (14,669)
Meeting guidelines	352 (28.5)
Not meeting guidelines	884 (71.5)
Daily fruit and vegetable intake, g, *N* = 1258	404 (318)
Meeting guidelines	577 (45.9)
Not meeting guidelines	681 (54.1)
Daily fibre intake, g, *N* = 1258	20 (8)
Meeting guidelines	125 (9.9)
Not meeting guidelines	1133 (90.1)
Weekly red meat intake, g, *N* = 1258	194 (280)
Meeting guidelines	1099 (87.4)
Not meeting guidelines	159 (12.6)
Daily processed meat intake, g, *N* = 1258	19 (32)
Meeting guidelines	628 (49.9)
Not meeting guidelines	630 (50.1)
Percentage of daily calories from free sugar, *N* = 1258	10.4 (5.3)
Meeting guidelines	181 (14.4)
Not meeting guidelines	1077 (85.6)
Percentage of daily calories from fat, *N* = 1258	35.6 (7.7)
Meeting guidelines	460 (36.6)
Not meeting guidelines	798 (63.4)
High calorie food, *N* = 1258	
Meeting guidelines	52 (4.1)
Not meeting guidelines	1206 (95.9)
Units of alcohol per week, *N* = 1314	6 (10)
Meeting guidelines	1138 (86.6)
Not meeting guidelines	176 (13.4)
Smoking, *N* = 1344	
Meeting guidelines	1294 (96.3)
Not meeting guidelines	50 (3.7)
Total CHBRI score, *N* = 1136	4.2 (1.3)

Values are presented as means (SD) for continuous variables and *n* (%) for categorical variables. *SD* standard deviation.

### Associations between anxiety/depression and CHBRI score

Experiencing anxiety/depression problems was not associated with CHBRI index scores after minimal adjustment for age and sex, and after full adjustment for age, sex, ethnicity, marital status, highest level of education, total number of comorbidities, cancer type, cancer stage, treatment, and time between cancer diagnosis and baseline assessments (*p* > 0.05) (Table 3).

**Table 3:** Cross-sectional association between anxiety/depression and CHBRI index in people living with and beyond breast, prostate, and colorectal cancer (*N* = 1348).

	B (95% CI)	*p*
Minimally adjusted^a^	− 0.10 (− 0.20, 0.00)	0.059
Adjusted^b^	− 0.07 (− 0.18, 0.03)	0.166

^a^Adjusted for age and sex.

^b^Adjusted for age, sex, ethnicity, marital status, highest level of education, total number of comorbidities, cancer type, cancer stage, treatment, and time between cancer diagnosis and baseline assessments.

### Associations between anxiety/depression and meeting WCRF guidelines

Experiencing anxiety/depression problems was associated with a 26% lower odds of meeting WCRF guidelines for average daily steps after adjustment for age and sex (95% CI 0.56, 0.98) (Table 4). The odds ratio was relatively unchanged after further adjustment for ethnicity, marital status, highest level of education, total number of comorbidities, cancer type, cancer stage, treatment, and time between cancer diagnosis and baseline assessments (OR = 0.73; 95% CI 0.55, 0.97). There were no associations between anxiety/depression and meeting WCRF guidelines for average weekly aerobic steps, diet, alcohol or smoking.

**Table 4:** Cross-sectional associations between anxiety/depression and meeting WCRF recommendations in people living with and beyond breast, prostate, and colorectal cancer (*N* = 1348).

	Minimally adjusted^a^	*p*	Fully adjusted^b^	*p*
Average daily steps	0.74 (0.56–0.98)	0.034*	0.73 (0.55–0.97)	0.031*
Average weekly aerobic steps	0.85 (0.71–1.01)	0.072	0.89 (0.74, 1.07)	0.216
Daily fruit and veg intake	0.93 (0.80–1.08)	0.329	0.96 (0.82–1.12)	0.583
Daily fibre intake	0.93 (0.72–1.21)	0.602	0.92 (0.70–1.21)	0.572
Weekly red meat intake	1.07 (0.85–1.36)	0.553	1.10 (0.86–1.39)	0.456
Daily processed meat intake	1.04 (0.89–1.21)	0.657	1.06 (0.90–1.24)	0.489
High calorie food	0.65 (0.40–1.04)	0.073	0.68 (0.42–1.10)	0.112
Units of alcohol per week	0.97 (0.78–1.20)	0.757	0.95 (0.75–1.19)	0.628
Smoking^c^	0.71 (0.51–0.98)	0.038	0.77 (0.55–1.08)	0.133

Results presented as odds ratios (95% CI).

*p < 0.05.

^a^Adjusted for age and sex.

^b^Adjusted for age, sex, ethnicity, marital status, highest level of education, total number of comorbidities, cancer type, cancer stage, treatment, and time between cancer diagnosis and baseline assessments.

^c^Reference category: non-smokers.

### Sensitivity analyses

In the sensitivity analysis on a sample of participants with no missing data on the exposure, outcome and covariates (*N* = 852), the findings were mostly consistent with the main analysis (Supplementary Tables 2 and 3). There was an association between anxiety/depression and average daily steps that was directionally consistent with associations found in the main analysis but did not reach statistical significance (*p* > 0.05). There was also an association between experiencing anxiety/depression problems and a lower odds for meeting WCRF guidelines for high calorie food after adjustment for age and sex (OR = 0.39; 95% CI 0.19, 0.78) and after multivariable adjustment (OR = 0.41; 95% CI 0.20, 0.83).

In the sensitivity analysis additionally adjusting for body mass index, the results were similar to the main analyses (Supplementary Tables 4 and 5).

## Discussion

This study found that approximately 40% of the sample of people LWBC were experiencing anxiety/depression problems. Participants were adhering to an average of four of the nine recommended health behaviours. Psychological distress was associated with not adhering to average daily step recommendations for people LWBC. This association persisted even after additionally adjusting for body mass index. Psychological distress was not associated with the total number of WCRF recommendations adhered to among people LWBC. Furthermore, psychological distress was not associated with meeting or not meeting recommendations for average weekly aerobic steps, diet, alcohol consumption or smoking.

Experiencing anxiety/depression problems was associated with not meeting WCRF recommendations for average daily steps among people LWBC. This finding is in line with theories of health behaviour such as the social ecological model which posit that psychological factors have an influence on health behaviours^22^. This finding is also in line with prior research showing that depression and anxiety are associated with lower levels of physical activity^23–25^ and not meeting physical activity recommendations in people LWBC^26,35^. However, our finding strengthens the existing evidence base through the use of device-assessed activity, a large sample size, and adjusting for multiple sociodemographic and health-related covariates, including body mass index. Sensitivity analyses on a sample of individuals who had no missing data on the exposure, outcome and covariates found no association between anxiety/depression and adherence to WCRF recommendations for average daily steps. This finding might be due to loss of power given the smaller sample size, or due to bias induced by missing data. One reason why anxiety/depression might decrease adherence to WCRF recommendations for average daily steps among people LWBC is that psychological distress can decrease motivation and energy levels which could make it harder to exercise^58^.

Our study found no association found between anxiety/depression problems and meeting WCRF recommendations for average weekly aerobic steps. This is despite finding an association with meeting WCRF recommendations for average daily steps. Prior research conducted in this cohort of people LWBC from the ASCOT trial has found that associations between physical activity and quality life/sleep differ depending on how physical activity is measured^40^. A previous study examining the association between depressive symptoms and physical activity in 201 people affected by breast cancer found that only changes in light- and moderate-intensity physical activity, but not vigorous-intensity physical activity, were associated with lower depressive symptom scores^23^. Therefore, it is possible that psychological distress is only associated with lighter physical activity (i.e. daily steps) rather than more vigorous activity (i.e. weekly aerobic steps). However, the previous study conducted in people with breast cancer looked at the association between psychological distress and physical activity in the opposite direction to our study. Another reason why there was no association found between distress and weekly aerobic steps in our study is that the measure of anxiety/depression used was a single item from the EQ-5D-5L which simply asked participants to report if they were “not”/”slightly”/”moderately”/”severely”/”extremely” anxious or depressed on that day. This measure did not capture specific symptoms of anxiety/depression, so self-report estimates might have been biased. This measure is also not a cancer-specific measure of distress, only captures feelings of anxiety/depression on the day of assessment and does not capture functional impairment resulting from distress. Future work should explore the association between psychological distress and meeting WCRF recommendations using other measures of distress.

This study also found that anxiety/depression was not associated with meeting WCRF recommendations for any dietary component, alcohol intake or smoking. These findings oppose prior research showing that anxiety/depression is associated with poorer diet^29,59^, higher alcohol intake and smoking^32–34^. However, one prior cross-sectional study of 255 people affected by various types of cancer from the Netherlands found that depressive symptoms was not associated with fruit or vegetable consumption, alcohol intake and smoking^30^. It is interesting that anxiety/depression was only associated with meeting WCRF recommendations for physical activity in this study, given that unhealthy behaviours tend to cluster within individuals^60^. One reason for the lack of associations found in this study between anxiety/depression and meeting WCRF recommendations for diet, alcohol and smoking could be the low proportion of people meeting/not meeting guidelines for certain behaviours. For instance, only 9.9% were meeting guidelines for daily fibre intake and 4.1% were meeting guidelines for high calorie food. These low proportions highlight the importance of promoting behaviour change throughout the cancer continuum. On the other hand, most people were meeting guidelines for weekly red meat intake (87.4%), units of alcohol per week (86.6%) and smoking (96.3%). Another reason for our null findings is that we used measures of adherence to WCRF recommendations as our outcomes, whereas prior research has used continuous measures of dietary components/alcohol intake. For instance, a cross-sectional study of 205 people with breast cancer from China found that those who were depressed had lower protein, fibre and overall diet quality compared to those who were not depressed^29^. Prior research has also used smaller sample sizes, relied on surveys to measure dietary intake rather than more accurate measures such as 24 h recalls, and tended to examine the association between psychological distress and diet/alcohol/smoking in the opposite direction^32,33^.

Overall, this study found that 42% of people affected by breast, prostate and colorectal cancer were experiencing anxiety/depression problems. This proportion is higher than reported by prior research. A study of 10,153 people with cancer found that the prevalence of subclinical levels of anxiety was 22.6% and 16.5% for depression^9^. A meta-analysis of 211 studies found that the pooled mean prevalence of depression in people with cancer ranged from 8 to 24%, depending on the type of instrument, cancer type and treatment phase^61^. One reason for the high prevalence levels of distress found in this study (as well as the lack of associations found between distress and meeting WCRF recommendations for diet, alcohol and smoking) is due to the measure of anxiety/depression used, as previously discussed. Despite the high prevalence of anxiety/depression among people LWBC, research shows that it tends to be under-detected and undertreated^62–64^. Thus, it is important for healthcare professionals to screen for and treat psychological distress in cancer care.

There are several strengths of this study. First, we analysed data from a large sample of people living with and beyond breast, prostate and colorectal cancer, three of the most commonly diagnosed cancers^65^. Second, we included an array of important sociodemographic and health-related confounders in analyses. Third, to reduce bias due to missing data, multiple imputation was used. However, there are also some limitations to note. First, this study was cross-sectional, meaning that cause-and-effect and directionality cannot be inferred between psychological distress and adherence to WCRF recommendations. Studies have shown that physical inactivity may lead to changes in depressive symptoms among people LWBC^23,66^. Nevertheless, there are several plausible mechanisms linking psychological distress and adherence to WCRF recommendations, as previously discussed. Cross-sectional data were used in this study as main trial analyses were still underway at the time of writing. Planned future work will explore longitudinal response to intervention and associations with cancer survival data. Second, the cohort was a sample of people who had signed up to take part in a trial of a lifestyle intervention so may not be representative of all people LWBC. Third, although CHBRI scores are useful as health behaviours tend to co-occur and cluster among people LWBC^14^, these scores are somewhat limited as they place equal weighting on all individual behaviours even though different behaviours have different associations with health outcomes (e.g. smoking vs. fibre). Fourth, the time between diagnosis of cancer and baseline assessments in this study was on average three years, meaning that anxiety/depression captured in this study may not have been due to being diagnosed with cancer. However, despite this long time period, there is extensive research showing that a cancer diagnosis contributes to psychological distress^61^. There is also evidence showing that the prevalence of distress remains high for many years following a cancer diagnosis^67^. Thus, it is likely that at least some of the anxiety/depression captured in this study was due to being diagnosed with cancer. Finally, this study consisted of people living with and beyond breast, prostate and colorectal cancer, even though cancer is a heterogeneous condition. Future work could explore whether the association between distress and adherence to WCRF recommendations differs by cancer type.

In conclusion, this study has shown that anxiety/depression is associated with not meeting WCRF recommendations for average daily steps in people LWBC. Not meeting physical activity recommendations may explain why anxiety/depression is associated with poorer survival in people LWBC. Future prospective research is needed to examine whether depression is associated with changes in meeting WCRF recommendations among people LWBC, and whether physical activity is a mechanism linking distress and poorer outcomes among people LWBC. Furthermore, future work is needed to elucidate the mechanisms linking anxiety/depression with meeting WCRF recommendations among people LWBC. Ultimately, detecting and treating psychological morbidity among people LWBC is crucial to improve outcomes.

### Supplementary Information

Supplementary Tables.

## Data Availability

The datasets used and/or analysed in this study are available from the corresponding author upon reasonable request.
