# Supplementary material for: Psychological distress and health behaviours in people living with and beyond cancer: a cross-sectional study
Source: Sci Rep. 2024 Jul 4;14:15367. doi: 10.1038/s41598-024-66269-6 (PMC11224398; doi:10.1038/s41598-024-66269-6)
Supplement: Supplementary file 1 — Supplementary Tables. [file 41598_2024_66269_MOESM1_ESM.docx]

**Supplementary Table 1.** Participant characteristics at baseline in observed vs. imputed data (*N* = 1348)

|  | Observed | Imputed |
| --- | --- | --- |
|  | Mean (SD)/*n* (%) | Mean (SD)/*n* (%)^a^ |
| Age, years, *N* = 1345 | 64.2 (11.4) | 64.2 (11.4) |
| Sex, *N* = 1348 |  |  |
| Male | 520 (38.6) | 520 (38.6) |
| Female | 828 (61.4) | 828 (61.4) |
| Ethnicity, *N* = 1342 |  |  |
| White | 1242 (92.5) | 1247 (92.5) |
| Non-white | 100 (7.5) | 101 (7.5) |
| Marital status, *N* = 1347 |  |  |
| Married | 957 (71.1) | 958 (71.0) |
| Not married | 390 (29.0) | 390 (29.0) |
| Highest education, *N* = 1254 |  |  |
| None | 226 (18.0) | 249 (18.4) |
| GCSE/Vocational | 412 (32.9) | 448 (33.2) |
| A level | 174 (13.9) | 187 (13.8) |
| Degree or above | 442 (35.2) | 465 (34.5) |
| Total number of comorbidities, *N* = 1348 | 0.8 (1.0) | 0.8 (1.0) |
| 0 | 643 (47.7) | 643 (47.7) |
| 1 | 436 (32.3) | 436 (32.3) |
| 2 | 176 (13.1) | 176 (13.1) |
| 3 | 70 (5.2) | 70 (5.2) |
| 4+ | 23 (1.6) | 23 (1.6) |
| Cancer type, *N* = 1348 |  |  |
| Breast | 711 (52.7) | 711 (52.7) |
| Prostate | 352 (26.1) | 352 (26.1) |
| Colorectal | 238 (17.7) | 238 (17.7) |
| Breast/prostate/colorectal + one other | 47 (3.5) | 47 (3.5) |
| Cancer stage, *N* = 1136 |  |  |
| 0 | 28 (2.5) | 34 (2.5) |
| 1 | 439 (38.6) | 519 (38.5) |
| 2 | 426 (37.5) | 504 (37.4) |
| 3 | 229 (20.2) | 274 (20.3) |
| 4 | 14 (1.23) | 17 (1.2) |
| Treatment, *N* = 1321 |  |  |
| Surgery only | 264 (20.0) | 270 (20.0) |
| Surgery and at least one other | 780 (59.1) | 791 (58.7) |
| Any combination of other treatment | 208 (15.8) | 215 (15.9) |
| No treatment or active surveillance | 69 (5.2) | 72 (5.3) |
| Time between cancer diagnosis and baseline assessments, days, *N* = 1348 | 1070.9 (382.1) | 1070.9 (382.1) |
| BMI, *N* = 1273 | 27.0 (4.7) | 27.0 (4.8) |
| EQ-5D-5L anxiety/depression severity, *N* = 1329 |  |  |
| No problems | 777 (58.5) | 787 (58.4) |
| One or more problems | 552 (41.5) | 561 (41.6) |
| Average daily steps, *N* = 1236 | 5905 (3287) | 5876 (3424) |
| Meeting guidelines | 133 (10.8) | 143 (10.7) |
| Not meeting guidelines | 1103 (89.24) | 1205 (89.4) |
| Average weekly aerobic steps, *N* = 1236 | 11539 (14669) | 11442 (15101) |
| Meeting guidelines | 352 (28.5) | 380 (28.2) |
| Not meeting guidelines | 884 (71.5) | 968 (71.8) |
| Daily fruit and vegetable intake, g, *N* = 1258 | 404 (318) | 403 (332) |
| Meeting guidelines | 577 (45.9) | 617 (45.8) |
| Not meeting guidelines | 681 (54.1) | 731 (54.2) |
| Daily fibre intake, g, *N* = 1258 | 20 (8) | 20 (8) |
| Meeting guidelines | 125 (9.9) | 133 (9.8) |
| Not meeting guidelines | 1133 (90.1) | 1215 (90.2) |
| Weekly red meat intake, g, *N* = 1258 | 194 (280) | 194 (287) |
| Meeting guidelines | 1099 (87.4) | 1178 (87.4) |
| Not meeting guidelines | 159 (12.6) | 170 (12.6) |
| Daily processed meat intake, g, *N* = 1258 | 19 (32) | 19 (34) |
| Meeting guidelines | 628 (49.9) | 671 (49.8) |
| Not meeting guidelines | 630 (50.1) | 677 (50.2) |
| Percentage of daily calories from free sugar, *N* = 1258 | 10.4 (5.3) | 10.4 (5.3) |
| Meeting guidelines | 181 (14.4) | 194 (14.4) |
| Not meeting guidelines | 1077 (85.6) | 1154 (85.6) |
| Percentage of daily calories from fat, *N* = 1258 | 35.6 (7.7) | 36 (7.7) |
| Meeting guidelines | 460 (36.6) | 492 (36.5) |
| Not meeting guidelines | 798 (63.4) | 856 (63.5) |
| High calorie food, *N* = 1258 |  |  |
| Meeting guidelines | 52 (4.1) | 55 (4.1) |
| Not meeting guidelines | 1206 (95.9) | 1293 (95.9) |
| Units of alcohol per week, *N* = 1314 | 6 (10) | 6 (10) |
| Meeting guidelines | 1138 (86.6) | 1166 (86.5) |
| Not meeting guidelines | 176 (13.4) | 181 (13.5) |
| Smoking, *N* = 1344 |  |  |
| Meeting guidelines | 1294 (96.3) | 1298 (96.3) |
| Not meeting guidelines | 50 (3.7) | 50 (3.7) |
| Total CHBRI score, *N* = 1136 | 4.2 (1.4) | 4.2 (1.3) |

*Note.* *SD* = standard deviation.

Values are presented as means (SD) for continuous variables and *n* (%) for categorical variables.

^a^ For imputed data, pooled *n*s and %s are shown across the 20 imputed datasets.

**Supplementary Table 2.** Cross-sectional association between anxiety/depression and CHBRI index in people living with and beyond breast, prostate, and colorectal cancer (*N* = 852; complete case analysis).

|  | B (95% CI) | *p* |
| --- | --- | --- |
| Minimally adjusted^a^ | -0.08 (-0.22, 0.05) | 0.242 |
| Adjusted^b^ | -0.07 (-0.20, 0.07) | 0.325 |

*Notes.* *p<0.05; **p<0.01, ***p<0.001.

^a^Adjusted for age and sex.

^b^Adjusted for age, sex, ethnicity, marital status, highest level of education, total number of comorbidities, cancer type, cancer stage, treatment, and time between cancer diagnosis and baseline assessments.

**Supplementary Table 3.** Cross-sectional associations between anxiety/depression and meeting WCRF recommendations in people living with and beyond breast, prostate, and colorectal cancer (complete case analysis).

|  | Minimally adjusted^a^ | *p* | Fully adjusted^b^ | *p* |
| --- | --- | --- | --- | --- |
| Average daily steps, *N* = 905 | 0.78 (0.57-1.05) | 0.105 | 0.75 (0.55-1.03) | 0.074 |
| Average weekly aerobic steps, *N* = 905 | 0.89 (0.73-1.09) | 0.252 | 0.92 (0.75-1.14) | 0.438 |
| Daily fruit and veg intake, *N* = 927 | 0.93 (0.78-1.12) | 0.453 | 0.96 (0.80-1.15) | 0.645 |
| Daily fibre intake, *N* = 927 | 0.93 (0.70-1.24) | 0.614 | 0.91 (0.68-1.22) | 0.521 |
| Weekly red meat intake, *N* = 927 | 0.96 (0.73-1.26) | 0.778 | 1.00 (0.76-1.33) | 0.972 |
| Daily processed meat intake, *N* = 927 | 1.10 (0.92-1.31) | 0.320 | 1.09 (0.91-1.32) | 0.339 |
| High calorie food, *N* = 927 | 0.39 (0.19-0.78) | 0.008** | 0.41 (0.20-0.83) | 0.013* |
| Units of alcohol per week, *N* = 972 | 0.86 (0.67-1.10) | 0.223 | 0.84 (0.65-1.08) | 0.178 |
| Smoking ^c^, *N* = 984 | 0.72 (0.48-1.07) | 0.101 | 0.81 (0.54-1.23) | 0.325 |

*Notes.* *p<0.05; **p<0.01, ***p<0.001.

Results presented as odds ratios (95% CI).

^a^Adjusted for age and sex.

^b^Adjusted for age, sex, ethnicity, marital status, highest level of education, total number of comorbidities, cancer type, cancer stage, treatment, and time between cancer diagnosis and baseline assessments.

^c^ Reference category: non-smokers.

**Supplementary Table 4.** Cross-sectional association between anxiety/depression and CHBRI index in people living with and beyond breast, prostate, and colorectal cancer, additionally adjusting for body mass index (*N*= 1348).

|  | B (95% CI) | *p* |
| --- | --- | --- |
| Minimally adjusted^a^ | -0.10 (-0.20, 0.00) | 0.059 |
| Adjusted^b^ | -0.05 (-0.15, 0.05) | 0.323 |

*Notes.* *p<0.05; **p<0.01, ***p<0.001.

^a^Adjusted for age and sex.

^b^Adjusted for age, sex, ethnicity, marital status, highest level of education, total number of comorbidities, cancer type, cancer stage, treatment, time between cancer diagnosis and baseline assessments and body mass index.

**Supplementary Table 5.** Cross-sectional associations between anxiety/depression and meeting WCRF recommendations in people living with and beyond breast, prostate, and colorectal cancer, additionally adjusting for body mass index (*N*= 1348).

|  | Minimally adjusted^a^ | *p* | Fully adjusted^b^ | *p* |
| --- | --- | --- | --- | --- |
| Average daily steps | 0.74 (0.56-0.98) | 0.034* | 0.75 (0.57-0.99) | 0.045* |
| Average weekly aerobic steps | 0.85 (0.71-1.01) | 0.072 | 0.91 (0.76-1.09) | 0.305 |
| Daily fruit and veg intake | 0.93 (0.80-1.08) | 0.329 | 0.97 (0.83-1.14) | 0.749 |
| Daily fibre intake | 0.93 (0.72-1.21) | 0.602 | 0.95 (0.72-1.25) | 0.706 |
| Weekly red meat intake | 1.07 (0.85-1.36) | 0.553 | 1.11 (0.87-1.41) | 0.398 |
| Daily processed meat intake | 1.04 (0.89-1.21) | 0.657 | 1.08 (0.92-1.27) | 0.323 |
| High calorie food | 0.65 (0.40-1.04) | 0.073 | 0.68 (0.42-1.10) | 0.114 |
| Units of alcohol per week | 0.97 (0.78-1.20) | 0.757 | 0.95 (0.76-1.19) | 0.649 |
| Smoking ^c^ | 0.71 (0.51-0.98) | 0.038 | 0.75 (0.54-1.06) | 0.102 |

*Notes.* *p<0.05; **p<0.01, ***p<0.001.

Results presented as odds ratios (95% CI).

^a^Adjusted for age and sex.

^b^Adjusted for age, sex, ethnicity, marital status, highest level of education, total number of comorbidities, cancer type, cancer stage, treatment, time between cancer diagnosis and baseline assessments and body mass index.

^c^ Reference category: non-smokers.
